# Sarcopenic obesity and history of falls in older Italian adults: associations according to different diagnostic combinations

**DOI:** 10.3389/fendo.2025.1727488

**Published:** 2026-01-13

**Authors:** Ana Lúcia Danielewicz, Gabrielle Freitas de Assis, Letícia Martins Cândido, Vanessa Amaral Mendonça, Ana Cristina Rodrigues Lacerda, Gabriella Tringali, Roberta De Micheli, Adele Bondesan, Núbia Carelli Pereira de Avelar, Alessandro Sartorio

**Affiliations:** 1 Department of Physiotherapy, Federal University of Santa Catarina, Araranguá, Santa Catarina, Brazil; 2 Graduate Program in Public Health, Federal University of Santa Catarina, Florianópolis, Santa Catarina, Brazil; 3 Department of Physiotherapy, Federal University of the Jequitinhonha and Mucuri Valleys, Diamantina, Minas Gerais, Brazil; 4 Istituto Auxologico Italiano, Istituto di Ricovero e Cura a Carattere Scientifico (IRCCS), Experimental Laboratory for Auxo-endocrinological Research, Milan, Italy

**Keywords:** aged, falls, obesity, older adults, sarcopenia

## Abstract

**Background:**

Sarcopenic obesity (SO) is characterized by the coexistence of sarcopenia and excess adipose tissue, increasing the risk of falls and related adverse outcomes in older adults. However, diagnostic criteria for SO remain inconsistent, warranting further investigation. This study aimed to examine the association between SO, defined according to the criteria proposed by the 2022 European Society for Clinical Nutrition and Metabolism (ESPEN) and the European Association for the Study of Obesity (EASO), and the history of falls among hospitalized older Italian adults.

**Methods:**

This cross-sectional study included 90 older Italian adults (≥ 60 years) with severe obesity (body mass index ≥ 35 kg/m^2^). The exposure variable, SO, was defined by the concomitant presence of reduced muscle function, high fat mass, and low muscle mass adjusted for body weight. Muscle function was assessed using the Five-Times Sit-to-Stand (5×STS) test and handgrip strength (HGS). Fat mass percentage (FM%) was measured using dual-energy X-ray absorptiometry (DXA) and bioelectrical impedance analysis (BIA). Appendicular muscle mass (AMM/W) and skeletal muscle mass (SMM/W), both adjusted for body weight, were assessed using DXA and BIA, respectively. Four diagnostic definitions of SO were analyzed according to the following combinations of tests and instruments: 1) 5×STS + FM% DXA + AMM/W, 2) 5×STS + FM% BIA + SMM/W, 3) HGS + FM% DXA + AMM/W, and 4) HGS + FM% BIA + SMM/W. The outcome was self-reported falls in the past year. Multivariable logistic regression models were adjusted for sex, age, education, marital status, fall-related multimorbidity, alcohol consumption, and regular physical activity.

**Results:**

Significant associations between SO and falls were found only when muscle function was assessed using HGS, regardless of the method used to estimate FM% and muscle mass. In adjusted analyses, participants classified as having SO according to combinations HGS + FM% BIA + SMM/W and HGS + FM% DXA + AMM/W had approximately almost fourfold (OR=3.61; 95% CI: 1.28–10.19) and sixfold (OR=6.03; 95% CI: 1.80–20.23) higher odds of reporting falls in the previous year, respectively, when compared to those without SO considering the same diagnostic tests.

**Conclusion:**

SO defined by low HGS combined with high FM% and low muscle mass adjusted for body weight—whether measured using DXA or BIA—was associated with greater odds of falls in older Italian adults. These findings support the use of muscle strength-based definitions of SO when evaluating fall risk in this population.

## 1 Introduction

Population aging is on the rise, and it is estimated that the number of older adults worldwide will triple by 2100 (1). In Europe, 21.3% of the population was aged 65 years or older in 2023, while in Italy, this proportion was 24.3% in 2024 (2). Considering that aging is a natural, dynamic, and progressive process that results in significant physiological, social, and psychological changes, older adults become more vulnerable and predisposed to health-impairing conditions (3).

Sarcopenia is a common condition in older adults (4), and according to the Global Leadership Initiative on Sarcopenia (GLIS), it is defined as a generalized skeletal muscle disease (5). In addition, it is associated with increased risks of falls, fractures, metabolic disorders, disabilities, and even higher mortality (6).

Another increasingly prevalent chronic condition among older adults is obesity, characterized by excessive body fat accumulation, which can negatively impact multiple body systems and contribute to the development of various chronic diseases (7). According to the World Health Organization, obesity is already considered a global epidemic and one of the greatest public health challenges of the century (7). In 2019, it was estimated that approximately half of the adult European population was overweight or obese, with a marked increase in the proportion of overweight individuals as age advanced (8). Among older adults, those aged 65 to 74 years showed the highest proportion of overweight individuals, totaling 63.6% (2).

In addition to the existence of these conditions in isolation, obesity and sarcopenia can coexist, a condition known as sarcopenic obesity (SO) (9). According to the most recent European Society for Clinical Nutrition and Metabolism/European Association for the Study of Obesity (ESPEN/EASO) consensus, published in 2022 (10), SO should be diagnosed based on excess body fat, considering elevated body mass index (BMI) or waist circumference values, concomitant with low muscle mass and function, as assessed using various physical tests and body composition measurement tools. This clinical condition combines the risks associated with both obesity and sarcopenia, resulting in higher rates of morbidity, functional disability, and risk of falls (11).

Moreover, the prevalence of SO increases with age, and it is estimated that more than 1 in 10 older adults worldwide is affected by this condition (11). The prevalence of SO in older adults varies according to the diagnostic methods used in studies. According to a previous study conducted by our research group, the prevalence of SO was 40% when using the skeletal muscle mass adjusted for body weight (SMM/W) index combined with the handgrip strength (HGS) test, and 23% when muscle mass was assessed using the appendicular lean mass adjusted for body weight (ALM/W) index along with the Five-Times Sit-to-Stand test (5×STS) (12). Another recent study of older women found a SO prevalence of 41% using dual-energy X-ray absorptiometry (DXA) and HGS as diagnostic methods (13).

Among the negative outcomes associated with SO, a high risk of falls has been observed, which is even greater when combined with other factors, including physiological changes associated with aging, increased prevalence of chronic diseases, reliance on walking aids, previous history of falls, and fear of falling (14). In a systematic review and meta-analysis, older adults with SO were found to have a 30% higher risk of falls compared to those without this condition (15).

Therefore, understanding how SO is associated with the risk of falls is essential to developing strategies to prevent this outcome, reduce related health problems, and contribute to lower costs associated with hospitalizations and post-fall fracture rehabilitation in healthcare systems. Accordingly, the objective of this study was to analyze the association between different diagnostic combinations of SO and the occurrence of falls among hospitalized older Italian adults with severe obesity, thereby providing relevant contributions to clinical practice and health interventions aimed at preventing these comorbidities.

## 2 Materials and methods

### 2.1 Study design, setting, participants, and ethical aspects

This was a cross-sectional study of 90 older Italian adults with severe obesity who were hospitalized between April and November 2023 in the Pulmonary Division and the Rehabilitation Medicine Division of the Istituto Auxologico Italiano, IRCCS, in Piancavallo (VB), Italy. All patients were admitted to a multidisciplinary body weight reduction program, which included a calorie-restricted diet, physical rehabilitation, psychological support, and nutritional education (16). Participants aged 60 years or older with a BMI of 35 kg/m^2^ or higher were included in the study. BMI was calculated using the standard formula: BMI=weight (kg)/[height (m)]^2^. Individuals with prostheses, those unable to ambulate, those with cardiopulmonary or metabolic conditions that prevented any physical exertion, and those with neurological or motor neuron dysfunctions were excluded.

The study was approved by the Ethics Committee of Istituto Auxologico Italiano, IRCCS, Milan, Italy (protocol code 01C313; date of approval March 21, 2023; ethical code number 2023_03_21_07; acronym, PREFISAR), and was conducted in accordance with the ethical principles established in the Declaration of Helsinki, version 2013. All participants provided written informed consent.

### 2.2 Exposure variable: sarcopenic obesity

**Figure 1:**
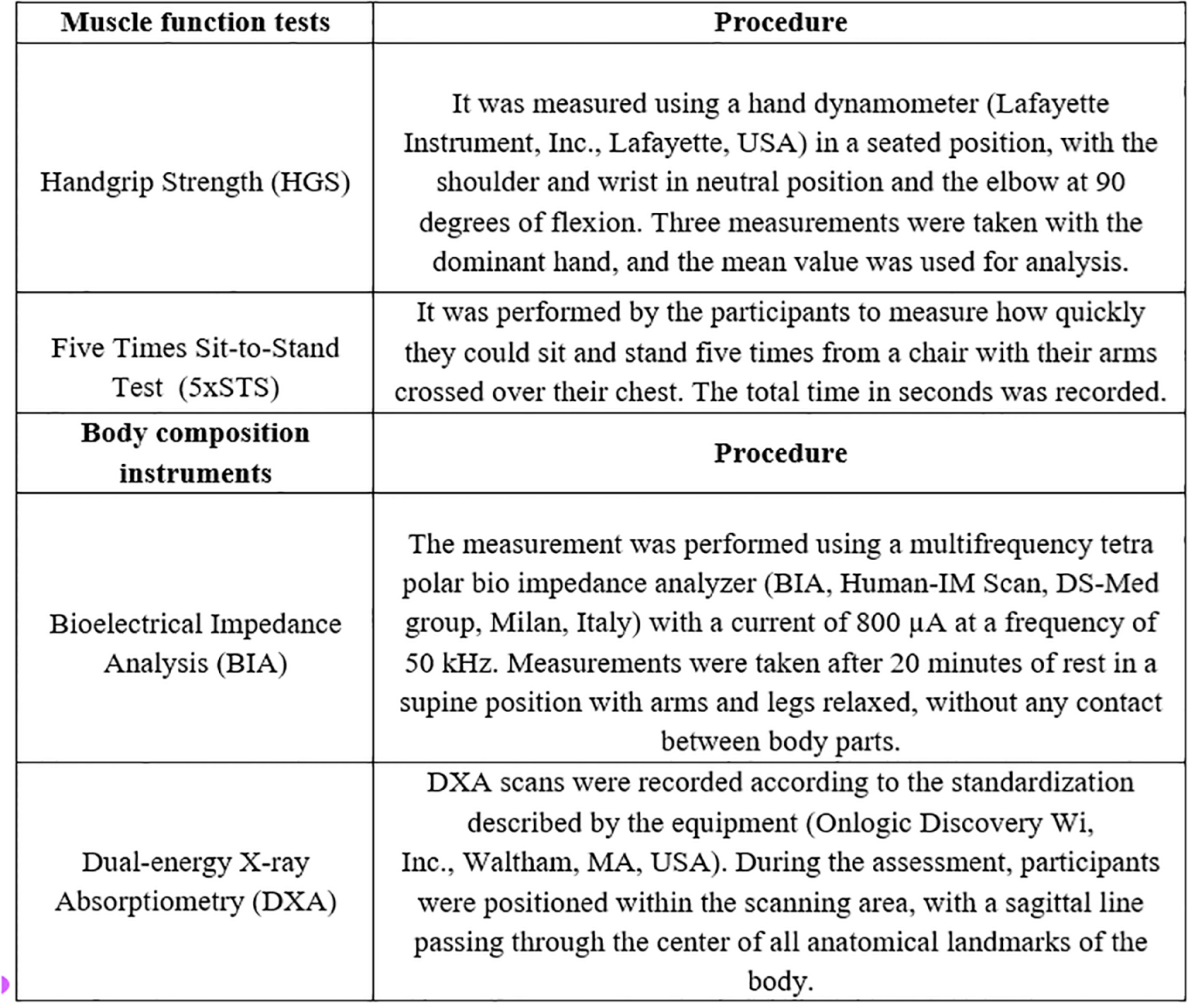
Procedures of muscle function tests and body composition instruments used with the study sample.

The assessment of SO was conducted according to the criteria of the 2022 ESPEN/EASO consensus (10). Using the different instruments described in **Figure 1**, the present study employed four SO diagnostic combinations for comparison:

- Assessment of SO using the 5×STS test and bioelectrical impedance analysis (BIA): low muscle function was evaluated using the 5×STS test, considering ≥17 seconds for both sexes. Body composition was assessed using BIA, taking into account excessive fat mass percentage (FM%: men >27.3%; women >40.7%) and low SMM/W (men <31.5%; women <22.1%) (17).
- Assessment of SO using the 5×STS test and DXA: low muscle function was evaluated using the 5×STS test, considering a cutoff of ≥ 17 seconds for both sexes. Body composition was measured using DXA, with the presence of excessive fat mass (FM%: men >31.0%; women >43.0%) and low appendicular muscle mass adjusted for body weight (AMM/W: men <25.7%; women <19.4%) (17).
- Assessment of SO using the HGS test and BIA: low muscle function was evaluated using the HGS test, considering <26 kg for men and <16 kg for women. Body composition was assessed using BIA, with excessive fat mass (men >27.3%; women >40.7%) and low SMM/W (men <31.5%; women <22.1%) (17).
- Assessment of SO using the HGS test and DXA: low muscle function was evaluated using the HGS test, considering values <26 kg for men and <16 kg for women. Body composition was measured using DXA, with the presence of excessive fat mass (men >31.0%; women >43.0%) and low appendicular lean mass adjusted for body weight (men <25.7%; women <19.4%) (17).

Exploratory analyses were additionally performed to assess SO based on low muscle mass adjusted for height squared, in accordance with the recommendations of the ESPEN/EASO consensus (10). Appendicular lean mass adjusted for height squared (ALM/height^2^) was derived from DXA measurements, and appendicular skeletal muscle mass adjusted for height squared (ASM/height^2^) was derived from BIA data. For both indicators, the cutoff points recommended by the European Working Group on Sarcopenia in Older People (EWGSOP2) (4) were applied, which define low muscle mass as <7.0 kg/m^2^ for men and <5.5 kg/m^2^ for women. Additionally, alternative cutoff strategies derived from the study sample were applied to define low muscle mass (z-score < −2 standard deviations and values below the 20th percentile).

### 2.3 Outcome variable: history of falls

The history of falls in the past 12 months was assessed using the question “Have you experienced any falls in the past year?” with response options of “yes” or “no”.

### 2.4 Adjustment variables

The following variables were adopted: sex (female or male), age group (60–69 or ≥ 70 years) (15), educational level (primary, secondary, or higher), marital status (single, divorced, married, or widowed), alcohol consumption (never, monthly or less, 2–4 times per month, or ≥ 4 times per week), self-reported fall-related multimorbidity (<2 chronic diseases or ≥ 2 chronic diseases; including back pain, arthritis, stroke, osteoporosis, labyrinthitis), and regular physical activity before hospitalization (yes or no).

### 2.5 Statistical analyses

Analyses were performed using the Stata statistical software, version 14.0 (Stata Corp., College Station, TX, USA). Descriptive analyses were performed, including the calculation of the prevalence (%) of the outcome according to the sample characterization variables. Associations between categorical variables were examined using Pearson’s chi-square test, considering p-values < 0.05 as statistically significant. To examine the associations between the different SO diagnostic combinations and history of falls, multivariable logistic regression analysis was performed, estimating crude and adjusted odds ratios (ORs) with 95% confidence intervals (95% CI). All analyses were conducted at a significance level of 5%.

## 3 Results

A total of 90 older adults with severe obesity were analyzed (median BMI: 43.2 kg/m^2^; interquartile range: 39.4–48.2), with the majority being women (52.2%) and aged 60–69 years (53.3%). The prevalence of falls was 41.1% in the total sample, with 57.4% among women and 23.2% among men. A significantly higher prevalence of falls was observed among women (57.5%; p=0.001) and among those with fall-related multimorbidity (53.0%; p=0.009) (**Table 1**).

**Table 1:** Sample description and prevalence of history of falls according to sociodemographic variables, lifestyle factors, health conditions, and sarcopenic obesity (SO) criteria in older Italian adults.

Variables	Total (N=90) n (%)	History of falls in the last 12 months		
		No (n=53) n (%)	Yes (n=37) n (%)	P-value
Sex				
Female	47 (52.2)	20 (42.5)	27 (57.5)	0.001*
Male	43 (47.8)	33 (76.7)	10 (23.3)	
Age group (years)				
60–69	48 (53.3)	31 (64.6)	17 (35.4)	0.240
≥70	42 (46.7)	22 (52.4)	20 (47.6)	
Educational level				
Primary education	26 (28.9)	12 (46.1)	14 (53.8)	0.272
Middle school	25 (27.8)	14 (56.0)	11 (44.0)	
High school	30 (33.3)	20 (66.7)	10 (33.3)	
Undergraduate	09 (10.0)	7 (77.8)	2 (22.2)	
Marital status				
Single/divorced	20 (22.2)	11 (55.0)	9 (45.0)	0.227
Married	22 (24.4)	10 (45.4)	12 (54.5)	
Widowed	48 (53.3)	32 (66.7)	16 (33.3)	
Alcohol use				
Never	42 (46.7)	23 (54.8)	19 (45.2)	0.555
Monthly or less	21 (23.3)	12 (57.1)	9 (42.9)	
≥2 times per month	14 (15.6)	8 (57.1)	6 (42.9)	
≥4 times per week	13 (14.4)	10 (76.9)	3 (23.1)	
Regular physical activity				
No	75 (83.3)	44 (58.7)	31 (41.3)	0.924
Yes	15 (16.7)	9 (60.0)	6 (40.0)	
Fall-related multimorbidity				
No	39 (43.3)	29 (74.3)	10 (25.7)	0.009*
Yes	51 (56.7)	24 (47.0)	27 (53.0)	
Sarcopenic obesity criteria				
5×STS + FM% BIA + SMM/W				
No	62 (68.9)	43 (69.3)	19 (30.6)	0.003*
Yes	28 (31.1)	10 (35.7)	18 (64.3)	
HGS + FM% BIA + SMM/W				
No	54 (60.0)	40 (74)	14 (25.9)	<0.001*
Yes	36 (40.0)	13 (36.1)	23 (63.9)	
HGS + FM% DXA + AMM/W				
No	67 (74.4)	46 (68.7)	21 (31.3)	0.001*
Yes	23 (25.6)	7 (30.4)	16 (69.6)	
5×STS + FM% DXA + AMM/W				
No	69 (76.7)	44 (63.7)	25 (36.2)	0.088
Yes	21 (23.3)	9 (42.8)	12 (57.1)	

5×STS, Five Times Sit-to-Stand; HGS, handgrip strength; FM%, fat mass percentage; AMM/W, appendicular muscle mass divided by body weight; SMM/W, skeletal muscle mass divided by body weight; DXA, dual-energy X-ray absorptiometry; BIA, bioelectrical impedance analysis.

*Significant p-value < 0.05 for Pearson’s chi-square test.

The proportion of our sample classified as having SO varied according to the diagnostic combinations applied. When the criteria included 5×STS, FM% (BIA), and SMM/W, 31.1% of participants met the definition of SO. The prevalence increased to 40.0% when HGS was combined with FM% (BIA) and SMM/W. In contrast, definitions incorporating DXA-based appendicular muscle mass resulted in lower prevalence estimates: 25.6% of participants met the criteria when HGS was combined with FM% (DXA) and AMM/W, and 23.3% when 5×STS was combined with FM% (DXA) and AMM/W. Further details are provided in **Table 1**.

Results from the additional exploratory analyses for the definition of SO, based on low appendicular muscle mass adjusted for height squared (ALM/height^2^ assessed using DXA and ASM/height^2^ assessed using BIA), indicated that no participant in our sample was classified as having SO, regardless of whether the EWGSOP2 cutoff points or alternative criteria derived from our sample (z-score < −2 standard deviations and values below the 20th percentile) were applied. These data are not shown in the tables.

**Figure 2:**
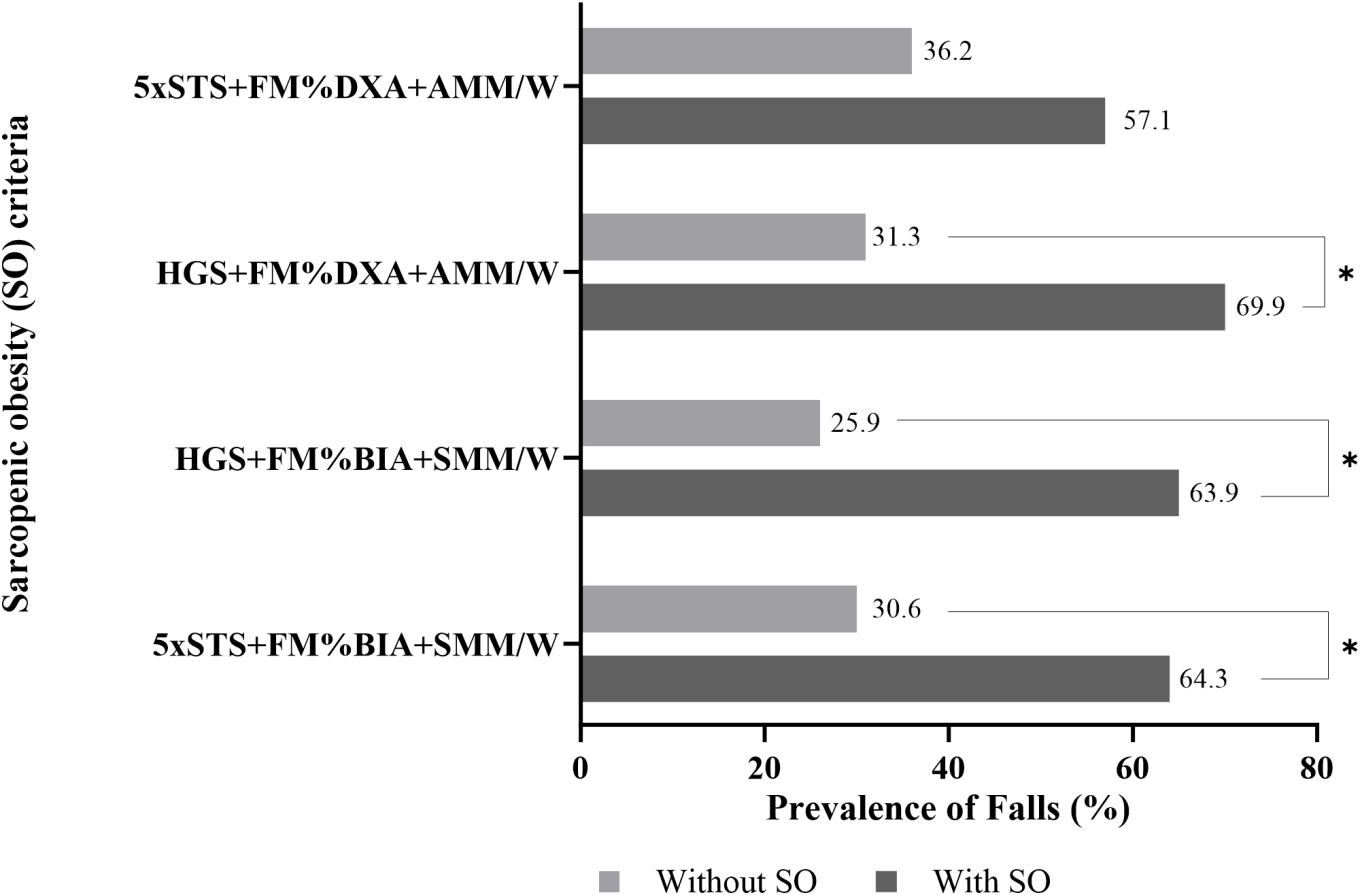
Prevalence of history of falls in older Italian adults with and without sarcopenic obesity (SO), based on different criteria. Legend: 5×STS, Five Times Sit-to-Stand; HGS, handgrip strength; FM%, fat mass percentage; AMM/W, appendicular muscle mass divided by body weight; SMM/W, skeletal muscle mass divided by body weight; DXA, dual-energy X-ray absorptiometry; BIA, bioelectrical impedance analysis. *Significant p-value <0.05 for Pearson’s chi-square test.

Statistically significant differences in the prevalence of falls were observed according to the presence or absence of SO across three of the four diagnostic criteria evaluated. The highest prevalence of falls (69.6%) was found in older adults with SO classified according to the HGS + FM% DXA + AMM/W criteria, followed by 64.3% according to the 5×STS + FM% BIA + SMM/W criteria, 63.9% according to the HGS + FM% BIA + SMM/W criteria, and 57.1% according to the 5×STS + FM% DXA + AMM/W criteria (**Figure 2**).

**Table 2** presents the results of the crude and adjusted logistic regression analyses examining the relationship between different SO diagnostic criteria and the occurrence of falls. Statistically significant associations were observed only when SO diagnoses were based on skeletal muscle function assessed using HGS, regardless of the body composition assessment method (DXA or BIA). Diagnoses of SO assessed using HGS + BIA and HGS + DXA increased the odds of falls in the evaluated elderly by almost fourfold (OR=3.61; 95% CI: 1.28–10.19) and sixfold (OR=6.03; 95% CI: 1.80–20.23), respectively, when compared to those without SO, considering the same diagnostic tests.

**Table 2:** Crude and adjusted logistic regression analyses of the association between sarcopenic obesity (SO), evaluated by different criteria, and history of falls in the last 12 months in older Italian adults (N=90).

Sarcopenic obesity criteria	History of falls in the last 12 months	
	Crude OR (95% CI)	Adjusted* OR (95% CI)
5×STS + FM% BIA + SMM/W	**4.07 (1.58; 10.45)**	2.80 (0.95; 8.29)
HGS + FM% BIA + SMM/W	**5.05 (2.02; 12.59)**	**3.61 (1.28; 10.19)**
HGS + FM% DXA + AMM/W	**5.00 (1.79; 13.98)**	**6.03 (1.80; 20.23)**
5×STS + FM% DXA + AMM/W	2.34 (0.86; 6.34)	2.18 (0.70; 6.71)

Statistically significant (p-value < 0.05) values are in bold.

5×STS, Five Times Sit-to-Stand; HGS, handgrip strength; FM%, fat mass percentage; AMM/W, appendicular muscle mass divided by body weight; SMM/W, skeletal muscle mass divided by body weight; DXA, dual-energy X-ray absorptiometry; BIA, bioelectrical impedance analysis; OR, odds ratio; 95% CI, 95% confidence interval.

*Adjusted for sex, age group, educational level, marital status, alcoholism, fall-related multimorbidity, and regular physical activity.

## 4 Discussion

The main findings of our study showed higher odds of a history of falls only when the SO diagnosis included muscle function assessed using HGS, regardless of the method used to evaluate fat mass and muscle mass. Specifically, the diagnosis of SO based on the combination of low HGS values, high fat mass percentage, and low muscle mass percentage identified by BIA was associated with an almost fourfold increase in the odds of falls, whereas the diagnosis of SO using the same combination assessed using DXA was associated with a sixfold increase in the odds of falls among the older adults analyzed, compared to those without SO. Furthermore, the prevalence of falls was significantly higher among older adults with SO compared to those without SO, according to three of the four diagnostic criteria adopted.

A recent systematic review (18) reported that the prevalence of falls among older adults with SO in the evaluated countries ranged from 29.47% to 60.53%. This variation was greater than that observed in our study, possibly due to the lack of standardized diagnostic methods and the non-adoption of the criteria recommended by EASO/ESPEN. In another study conducted among postmenopausal women, SO was associated with an increased risk of falls in the 65–79-year age group (Relative Risk (RR)=1.21, 95% CI: 1.05–1.39) (19).

The pathophysiology of SO is a multifactorial process involving a series of interconnected mechanisms that affect both adipose and muscle tissue. Key mechanisms include chronic inflammation, insulin resistance, oxidative stress, and age-related hormonal changes (20). Oxidative stress contributes to cellular damage and reduced muscle quality (21), while the decline in anabolic hormone levels, such as testosterone and Growth hormone (GH), further aggravates the condition (22). As a result, obesity and sarcopenia together create a vicious cycle: excess fat not only overloads the body but also stimulates inflammatory and catabolic processes that weaken muscles, thereby increasing the risk of falls and other complications in older adults (21).

The results of our study corroborate those of Li et al. (2024) (23), who reported a significant association between SO and history of falls (OR=1.61; 95% CI: 1.08–2.40) using the ESPEN/EASO criteria in a large multicenter cohort of community-dwelling older adults in China. Similarly, in our sample, diagnoses of SO based on low HGS values showed strong associations, increasing the odds of falls by nearly fourfold and sixfold, depending on the instrument used to assess fat mass and muscle mass (BIA or DXA).

Furthermore, descriptive results from Li et al. (2024) (23) revealed significant differences in HGS values between older adults with and without a history of falls (men: p=0.0246; women: p=0.0329), whereas body composition variables such as SMM/W, SMM/BMI, and FM showed no significant differences (p > 0.45). Taken together with our findings, these results highlight the central role of muscle function, as assessed using HGS, as the main component associated with fall risk in individuals with SO. Moreover, it has been reported that SO affects nearly 1 in 10 older adults worldwide, with prevalence varying substantially depending on the diagnostic criteria applied (24). Consistently, findings from a recent review indicated that the prevalence of SO is directly influenced by the diagnostic criteria adopted (25).

As in our study, HGS was used to define SO in the study by Montalvão-Sousa et al. (2024) (13), which also identified a significant association between SO defined according to ESPEN/EASO criteria and a higher risk of falls in older Brazilian women. In that study, the presence of SO was associated with a higher prevalence of falls in the previous year (53.5% in the SO group vs. 27% in the eutrophic group; p=0.045) and a higher estimated risk of falls (p=0.004). In contrast, Scott et al. (36) conducted a 5-year prospective cohort study of community-dwelling residents in southern Tasmania, in which muscle strength was assessed using isometric lower-limb strength testing. In this study, no significant association was observed between SO and fall risk. These findings suggest that HGS may be a more sensitive and discriminative marker for identifying individuals with greater functional vulnerability and fall risk, possibly because it better reflects overall strength and functional capacity (26).

Other studies have examined differences in functional outcomes when comparing HGS and the 5×STS in older populations. Low concordance between these tests when identifying probable sarcopenia suggests that they capture distinct physiological domains (27, 28). Longitudinal evidence also indicates a clear dose–response relationship between higher HGS and lower risk of falls in community-dwelling older adults (29). Although both tests assess aspects of muscle function, the 5×STS primarily reflects overall physical performance and is influenced by multiple factors such as trunk control, pain, lower-limb strength, dynamic balance, and cardiorespiratory capacity, which increases variability among older adults, particularly those who are hospitalized (30). In contrast, HGS is a reliable marker of global muscle strength, neuromuscular integrity, and physiological reserve, capturing broader dimensions of physical robustness. This may help explain the associations observed in our study and reinforces the importance of prioritizing HGS in diagnostic definitions of SO (31, 32).

The stronger association between DXA-defined SO and falls may reflect intrinsic differences in measurement accuracy between DXA and BIA. Evidence indicates that BIA often overestimates fat-free mass relative to DXA in individuals with excess adiposity, largely because of assumptions about hydration, tissue conductivity, and body geometry that do not hold in obese populations (33, 34). A recent systematic review (35) that included older Italian adults further confirmed that BIA tends to yield higher lean mass estimates than DXA in aging populations. Such overestimation can mask true muscle deficits, leading to misclassification of sarcopenia and attenuating observed risk estimates. This methodological limitation likely contributed to the weaker associations observed for BIA-defined SO in our study.

It is important to acknowledge the limitations of this study. The research was conducted on a small and specific sample (hospitalized older Italian adults with severe obesity), which may not reflect the realities of other populations and therefore limits the generalizability of the results. Although this was a cross-sectional analysis, all participants were already hospitalized due to severe obesity, making it highly likely that the reported falls occurred after the onset of SO, which aligns with our hypothesis of a causal relationship. Finally, the analyses were adjusted for potential confounders, thereby strengthening the validity of the findings and providing valuable insights to guide clinical strategies to prevent falls in this population.

Therefore, further studies are recommended to better explore the associations between SO and history of falls in older adults, both in hospitalized populations and in other settings, such as the community. Our findings also reinforce that the use of standardized and easily applicable diagnostic criteria, such as the handgrip strength test, can help identify older adults with SO who are at risk of falls. This, in turn, enables the development of targeted interventions to preserve and enhance muscle strength, thereby reducing the risk of falls, fractures, and related complications. Moreover, it can inform clinical protocols, rehabilitation strategies, and preventive actions, ultimately promoting a higher quality of life, greater autonomy, and lower hospitalization costs.

## 5 Conclusions

The study concluded that the assessment of muscle function, particularly through HGS, emerged as the most important component for diagnosing SO for identifying fall risk in hospitalized older Italian adults with severe obesity. The association between SO and a history of falls was significantly higher when HGS was incorporated into the diagnostic criteria, increasing the odds of falls by up to sixfold.

Our results, therefore, reinforce the need to adopt standardized diagnostic criteria, such as those proposed by ESPEN/EASO, which emphasize the assessment of muscle function using HGS as a practical and low-cost clinical marker. Implementing HGS as a diagnostic tool for SO may help identify older adults at higher risk of falls and support interventions focused on preserving muscle strength. Despite the study’s limitations, the findings provide an important contribution to the diagnosis of SO in hospitalized older adults. Although further studies are needed to confirm our results, current evidence suggests that muscle function assessment should be integrated into clinical protocols to prevent falls and their negative consequences.

## Data Availability

The datasets presented in this study can be found in online repositories. The names of the repository/repositories and accession number(s) can be found below: The raw dataset will be deposited in Zenodo.org upon acceptance of the article.

## References

[1] United Nations Regional Information C United Nations Regional Information Centre (UNRIC) . Ageing. Brussels: UNRIC;. Available online at: https://unric.org/pt/envelhecimento/ (Accessed October 12, 2025).

[2] Eurostat . Over half of adults in the EU are overweight. World Health Organization. World report on ageing and health. Geneva: WHO (2015). Available online at: http://www.who.int (Accessed October 12, 2025).

[3] World Health O . World report on ageing and health. Geneva: WHO (2015). Available online at: http://www.who.int (Accessed October 12, 2025).

[4] Cruz-Jentoft AJ . Sarcopenia: revised European consensus on definition and diagnosis. Age Ageing. (2019) 48:16–31. doi: 10.1093/ageing/afy169, PMID: 30312372 PMC6322506

[5] Kirk B . The conceptual definition of sarcopenia: delphi consensus from the global leadership initiative in sarcopenia (GLIS). Age Ageing. (2024) 53:afae052. doi: 10.1093/ageing/afae052, PMID: 38520141 PMC10960072

[6] Yuan S Larsson SC . Epidemiology of sarcopenia: prevalence. Risk Factors Consequences. (2023) 144:155533. doi: 10.1016/j.metabol.2023.155533, PMID: 36907247

[7] World Health O . Obesity. Geneva: World Health Organization (2025). Available online at: https://www.who.int/health-topics/obesity (Accessed October 12, 2025).

[8] European Commission E . Population structure and ageing. European Commission, Eurostat. Population structure and ageing. Luxembourg: European Commission (2025). Available online at: https://ec.europa.eu/eurostat/statistics-explained/index.php?title=Population_structure_and_ageing (Accessed October 12, 2025).

[9] Barazzoni R Bischoff SC Boirie Y . Sarcopenic obesity: time to meet the challenge. Clin. Nutr. (2018) 37:1787–93. doi: 10.1016/j.clnu.2018.04.018, PMID: 29857921

[10] Donini LM . Definition and diagnostic criteria for sarcopenic obesity: ESPEN and EASO consensus statement. Clin. Nutr. (2022) 41:990–1000. doi: 10.1016/j.clnu.2021.11.014, PMID: 35227529

[11] Ji T Li Y Ma L . Sarcopenic obesity: an emerging public health problem. Aging Dis. (2022) 13:379–88. doi: 10.14336/AD.2021.1006, PMID: 35371597 PMC8947824

[12] Danielewicz AL . Analysis of sarcopenic obesity prevalence and diagnostic agreement according to the 2022 ESPEN and EASO Consensus in hospitalized older adults with severe obesity. Front. Endocrinol. (2024) 15:1366229. doi: 10.3389/fendo.2024.1366229, PMID: 38966224 PMC11222587

[13] Montalvão-Sousa TM Ferreira PA Colombelli NL de Carvalho KMB Blazevich AJ Lima RM . Sarcopenic obesity defined by the ESPEN and EASO consensus statement in older women: risk of falls and bone mineral density implications. Arch Gerontol Geriatr. (2024) 124:105444. doi: 10.1016/j.archger.2024.105444, PMID: 38643667

[14] Hacıdursunoğlu Erbaş D Çınar F Eti Aslan F . Elderly patients and falls: a systematic review and meta-analysis. Aging Clin Exp Res. (2021) 33:2953–66. doi: 10.1007/s40520-021-01843-w, PMID: 33864235

[15] Gandham A . Falls, fractures, and areal bone mineral density in older adults with sarcopenic obesity: A systematic review and meta-analysis. Obes. Rev. (2021) 22:e13187. doi: 10.1111/obr.13187, PMID: 33491333

[16] Guerrini Usubini A . A three-week in-hospital multidisciplinary body weight reduction program exerts beneficial effects on physical and mental health and fatiguability of elderly patients with obesity. Front. Aging Neurosci. (2022) 14:1054941. doi: 10.3389/fnagi.2022.1054941, PMID: 36589548 PMC9800933

[17] Gortan Cappellari G . Sarcopenic obesity research perspectives outlined by the sarcopenic obesity global leadership initiative (SOGLI) – proceedings from the SOGLI consortium meeting in Rome November 2022. Clin. Nutr. (2023) 42:687–99. doi: 10.1016/j.clnu.2023.02.018, PMID: 36947988

[18] de Queiroz Júnior JRA dos Santos AKB da Costa BA dos Santos Calado GH Melo IO Barbosa JIL . Influência da obesidade sarcopênica no risco de quedas em idosos: uma revisão sistemática. Estudos Interdisciplinares Sobre o Envelhecimento. (2023) 28. doi: 10.22456/2316-2171.128536

[19] Follis S . Association between sarcopenic obesity and falls in a multiethnic cohort of postmenopausal women. J. Am. Geriatr. Soc. Int. (2018) 66:2314–20. doi: 10.1111/jgs.15613, PMID: 30375641 PMC6289680

[20] Wei S Nguyen TT Zhang Y Ryu D Gariani K . Sarcopenic obesity: epidemiology, pathophysiology, cardiovascular disease, mortality, and management. Front endocrinol. (2023) 14:1185221. doi: 10.3389/fendo.2023.1185221, PMID: 37455897 PMC10344359

[21] Gonzalez A Simon F Achiardi O Vilos C Cabrera D Cabello-Verrugio C . The critical role of oxidative stress in sarcopenic obesity. Oxid Med Cell Longev. (2021) 2021:4493817. doi: 10.1155/2021/4493817, PMID: 34676021 PMC8526202

[22] Hong S-h Choi KM . Sarcopenic obesity, insulin resistance, and their implications in cardiovascular and metabolic consequences. J. Mol. Sci. (2020) 21:494. doi: 10.3390/ijms21020494, PMID: 31941015 PMC7013734

[23] Li R Chen X Tang H Luo S Lian R Zhang W . Sarcopenic obesity and falls in older adults: A validation study of ESPEN/EASO criteria and modifications in Western China communities. Arch Gerontol Geriatr. (2024) 127:105557. doi: 10.1016/j.archger.2024.105557, PMID: 38964054

[24] Gao Q . Global prevalence of sarcopenic obesity in older adults: A systematic review and meta-analysis. Clin. Nutr. (2021) 40:4633–41. doi: 10.1016/j.clnu.2021.06.009, PMID: 34229269

[25] Luo Y Wang Y Tang S Xu L Zhao X Han M . Prevalence of sarcopenic obesity in the older non-hospitalized population: a systematic review and meta-analysis. BMC Geriatr. (2024) 24:357. doi: 10.1186/s12877-024-04952-z, PMID: 38649825 PMC11036751

[26] Tomkinson GR Lang JJ Rubín L McGrath R Gower B Boyle T . International norms for adult handgrip strength: A systematic review of data on 2.4 million adults aged 20 to 100+ years from 69 countries and regions. J Sport Health Sci. (2024) 14:101014. doi: 10.1016/j.jshs.2024.101014, PMID: 39647778 PMC11863340

[27] Núñez-Cortés R Andersen LL Cruz-Montecinos C Polo-López A López-Bueno R Calatayud J . Five-repetition chair-stand test vs. handgrip strength: Which better predicts mortality risk? A follow-up study in 43,605 middle-aged and older adults. Braz J Phys Ther. (2025) 29:101529. doi: 10.1016/j.bjpt.2025.101529, PMID: 40992236 PMC12492024

[28] Verstraeten LMG de Haan NJ Verbeet E van Wijngaarden JP Meskers CGM Maier AB . Handgrip strength rather than chair stand test should be used to diagnose sarcopenia in geriatric rehabilitation inpatients: REStORing health of acutely unwell adults (RESORT). Age Ageing. (2022) 51:afac242. doi: 10.1093/ageing/afac242, PMID: 36413590 PMC9681126

[29] Guo T Zhang F Xiong L Huang Z Zhang X Wan J . Association of handgrip strength with hip fracture and falls in community-dwelling middle-aged and older adults: A 4-year longitudinal study. Orthop Surg. (2024) 16:1051–63. doi: 10.1111/os.14029, PMID: 38485456 PMC11062856

[30] Yee XS Ng YS Allen JC Latib A Tay EL Abu Bakar HM . Performance on sit-to-stand tests in relation to measures of functional fitness and sarcopenia diagnosis in community-dwelling older adults. Eur Rev Aging Phys Act. (2021) 18:1. doi: 10.1186/s11556-020-00255-5, PMID: 33419399 PMC7791746

[31] Szaflik P Zadoń H Michnik R Nowakowska-Lipiec K . Handgrip strength as an indicator of overall strength and functional performance—Systematic review. Appl Sci. (2025) 15:1847. doi: 10.3390/app15041847

[32] Muñoz-Bermejo L Adsuar JC Mendoza-Muñoz M Barrios-Fernández S Garcia-Gordillo MA Pérez-Gómez J . Test-retest reliability of five times sit to stand test (FTSST) in adults: A systematic review and meta-analysis. Biol (Basel). (2021) 10:6510. doi: 10.3390/biology10060510, PMID: 34207604 PMC8228261

[33] Cheng KY Chow SK Hung VW Wong CH Wong RM Tsang CS . Diagnosis of sarcopenia by evaluating skeletal muscle mass by adjusted bioimpedance analysis validated with dual-energy X-ray absorptiometry. J Cachexia Sarcopenia Muscle. (2021) 12:2163–73. doi: 10.1002/jcsm.12825, PMID: 34609065 PMC8718029

[34] Lopes S Fontes T Tavares RG Rodrigues LM Ferreira-Pêgo C . Bioimpedance and dual-energy X-ray absorptiometry are not equivalent technologies: Comparing fat mass and fat-free mass. Int J Environ Res Public Health. (2022) 19:13940. doi: 10.3390/ijerph192113940, PMID: 36360820 PMC9657485

[35] Mandalà C Veronese N Dominguez LJ Candore G Accardi G Smith L . Use of bioelectrical impedance analysis in centenarians: a systematic review. Aging Clin Exp Res. (2023) 35:1–7. doi: 10.1007/s40520-022-02282-x, PMID: 36287324 PMC9816227

[36] Scott D . Associations between dietary nutrient intake and muscle mass and strength i n community-dwelling older adults: the Tasmanian Older Adult Cohort Study. Journal of the American Geriatrics Society. (2010) 58:2129–2134. doi: 10.1111/j.1532-5415.2010.03147.x, PMID: 21054294

